# Echocardiographic evaluation of working dogs of the Military Police of Rio de Janeiro: effects of the breed and body weight

**DOI:** 10.29374/2527-2179.bjvm001322

**Published:** 2022-06-28

**Authors:** Alex Moreira de Lima, Rodrigo Mencalha Moreira, Marcelo Salvador Gomes, Marcia Torres Ramos, Carlos Augusto dos Santos-Sousa, Paulo Souza-Júnior, Marcelo Abidu-Figueiredo

**Affiliations:** 1 Veterinarian, MSc. Autonomous, Rio de Janeiro, RJ, Brazil.; 2 Veterinarian, DSc. Autonomous, Rio de Janeiro, RJ, Brazil.; 3 Veterinarian, DSc. Faculdade de Medicina Veterinária. Universidade Estácio de Sá (UNESA). Niterói, RJ, Brazil.; 4 Veterinarian, DSc. Centro de Ciências Biológicas e da Natureza, Universidade Federal do Acre (UFAC). Rio Branco, AC, Brazil.; 5 Veterinarian, DSc. Departamento de Medicina Veterinária, Universidade Federal do Pampa (UNIPAMPA). Uruguaiana, RS, Brazil.; 6 Veterinarian, DSc. Departamento de Anatomia Animal e Humana, Universidade Federal Rural do Rio de Janeiro (UFRRJ). Seropédica, RJ, Brazil.

**Keywords:** cardiac ultrasound, echocardiogram, heart, patrol dogs, ultrassom cardíaco, ecocardiograma, coração, cães de patrulha

## Abstract

The Military Police of Rio de Janeiro state use dogs as a decisive tool for patrol and detection of drugs, weapons, and explosives. Complementary tests, such as echocardiography, are essential to maintain the integrity of these animals. This study aimed to evaluate the echocardiographic parameters of the working dogs belonging to the Military Police of Rio de Janeiro and compare them with the available data. Echocardiographic evaluation was performed on 48 healthy adult dogs from the Canine Action Battalion of the Military Police of Rio de Janeiro, Brazil. The sample consisted of 13 Labrador Retrievers, 12 Malinois Belgian Shepherds, 10 German Shepherds, 8 Dobermann Pinschers, and 5 Dutch Shepherds. Echocardiographic variables were correlated with body weight (BW). A positive correlation (P=0.0142, r=0.6837) between BW and the diameter of the left atrium was found in Malinois Belgian Shepherds. In German Shepherds, a positive correlation between BW and the internal diameter of the right ventricle during diastole (P=0.0320, r=0.6757) was observed; in addition, a positive correlation between BW and left ventricular internal diameter (P=0.0344, r=0.6689) during diastole was also found. Echocardiographic evaluations of these working dogs differed slightly from those previously established for similar-sized dog breeds.

## Resumo

No Rio de Janeiro, a Polícia Militar usa cães para patrulhar e detectar rotineiramente drogas, armas e explosivos. A realização de testes complementares como ecocardiogramas, é importante para manter a integridade dos animais. O objetivo deste estudo foi avaliar os parâmetros ecocardiográficos de cães trabalhadores da Polícia Militar do Rio de Janeiro e compará-los com os dados disponíveis na literatura. A avaliação ecocardiográfica foi realizada em quarenta e oito cães adultos saudáveis do Batalhão de Ação Canina da Polícia Militar do Rio de Janeiro. A amostra consistia de 13 Retriever de Labrador, 12 Pastores Belgas de Malinois, 10 Pastores Alemães, 8 Dobermann Pinschers e 5 Pastores Holandeses. Todas as variáveis ecocardiográficas foram correlacionadas com o peso corporal (PC). Foi encontrada uma correlação positiva (P=0,0142, r=0,6837) entre o peso e o diâmetro do átrio esquerdo nos Pastores Belgas de Malinois. Foi observada uma correlação positiva (P=0,0320, r= 0,6757) entre o peso corporal e o diâmetro interno do ventrículo direito durante a diástole para os pastores alemães. Além disso, foi encontrada uma correlação positiva (P= 0,0344, r=0,6689) entre o PC e o diâmetro interno do ventrículo esquerdo durante a diástole para Pastores Alemães ou Holandeses. As avaliações ecocardiográficas desses cães de trabalho diferiram ligeiramente dos padrões estabelecidos na literatura para raças porte semelhantes.

## Introduction

Echocardiography has been used in veterinary medicine since the early 1980s as a non-invasive method for assessing cardiac anatomy and function, providing quantitative information during systole and diastole, and serving as a tool for calculating myocardial function (Boon, 2011; Corda et al., 2019; Dickson et al., 2017; Tilley & Goodwin, 2002). It also allows the evaluation of the relationship between the structure of the heart and blood flow through direct visualization of the cardiac chambers (Corda et al., 2019; Dickson et al., 2017; Gugjoo et al., 2014; Vurucu et al., 2021).

Several echocardiographic indices have been used to evaluate ventricular function. The main limitation of their use is the variation among breeds of different somatotypes that warrants the need for appropriate values for each racial group (Koch et al., 1996; Morrison et al., 1992; Vurucu et al., 2021). In addition, body weight (BW), body surface area, sex, and age influence echocardiographic indices (Boon et al., 1983; Crippa et al., 1992; Dickson et al., 2017; Muzzi et al., 2006; Vurucu et al., 2021) as well as other factors, such as ventricular geometry, heart weight, and chest conformation (Dickson et al., 2017; Morrison et al., 1992; Snyder et al., 1995).

In Rio de Janeiro, the Military Police (PMERJ) use dogs to patrol and detect drugs, weapons, and explosives. These patrol dogs are housed at the Canine Action Battalion of the PMERJ kennels (BAC-PMERJ), and are trained for at least 50 min, divided into several sessions based on the training protocols. Although these animals undergo routine check-ups, cardiovascular preventive assessments are not included in the management of kennels. Therefore, it is essential to conduct further investigations, including echocardiographic evaluations, to ensure that the physical integrity of the animals is maintained. Furthermore, as many variables can significantly influence echocardiographic parameters, attention is necessary for echocardiographic measurements in different breeds.

This study aimed to evaluate the echocardiographic parameters of working dogs of the PMERJ and compare them with data available in the literature.

## Materials and methods

This study was approved by the BAC-PMERJ kennels and published in internal bulletin No. 084 (May 9^th^, 2012). This study was approved by the Ethics and Research Committee of the Universidade Federal Rural do Rio de Janeiro, No. 23083.002684 / 2012.59, and was conducted at the kennels of the BAC-PMERJ, located in Olaria, Rio de Janeiro, in 2015.

### Dogs

Of the 65 dogs housed at the BAC-PMERJ, 17 were excluded from the study because of the following exclusion criteria: overweight dogs; abnormal hematological or biochemical parameters; clinical, electrocardiographic, and echocardiographic signs of heart disease; or those who tested positive on *SNAP* test for *Dirofilaria immitis* antigens, *Anaplasma phagocytophilum*, *Anaplasma platys*, *Borrelia burgdorferi*, *Ehrlichia canis,* and *Ehrlichia ewingii* antibodies. Thus, for convenience, 48 dogs were included in this study: 13 Labrador Retrievers, 12 Malinois Belgian Shepherds, 10 German Shepherds, 8 Dobermann Pinschers, and 5 Dutch Shepherds. The dogs were divided into five groups according to their breed. According to the training protocols, these patrol dogs were between 3 and 5 years old and trained for at least 50 min throughout the day, divided into several sessions. All animals underwent clinical evaluations after 48 h of rest, involving cardiovascular assessment by electrocardiographic and echocardiographic examinations with color Doppler, laryngotracheal and cardiac auscultation, blood pressure measurement, oral and ocular mucosa assessment, rectal temperature measurement, thoracic circumference (TC) measurements, and blood and biochemical evaluation.

### Echocardiographic examination

Examination was performed using portable ultrasound equipment (Sono Site Titan®) with two-dimensional (2D) and M-mode echocardiography. The transducer used was a micro convex 5–8 MHz. Dogs were placed in the left lateral decubitus position with the transducer over the chest wall to obtain images through the right parasternal and left parasternal cranial and caudal windows using 2D and M-mode echocardiography. A thick acoustic gel layer was applied between the skin of the animal and the transducer to reduce air interference. The obtained M-mode parameters were evaluated and calculated according to Boon (1998) and Yamato et al. (2006). Variables measured in M-mode in the parasternal long-axis view at the level of the left ventricle were the right ventricular internal dimension in diastole (RVIDd), right ventricular internal dimension in systole (RVIDs), left ventricular internal dimension in diastole (LVIDd), left ventricular internal dimension in systole (LVIDs), interventricular septum thickness in diastole (ISTd), interventricular septum thickness in systole (ISTs), left ventricular free wall thickness in diastole (LVWTd), left ventricular free wall thickness in systole (LVWTs), aortic root dimensions (Ao), and left atrium dimensions (LA). Based on these measurements, the left atrial/aortic root ratio (LA/Ao), fractional shortening (FS), and left ventricular ejection fraction (LVEF) were calculated.

### Statistical analyses

The Shapiro–Wilk normality test was performed which resulted in a normal distribution of the variables. All measurements obtained by echocardiography were expressed as mean ± standard deviation (SD). Analysis of variance (ANOVA) with a post-hoc Tukey test was performed on the echocardiographic variables of the five studied dog breeds. In addition, Pearson's linear correlation was used to estimate the relationship between the animal’s BW and echocardiographic variables. The significance level was set at P < 0.05. Analyses were performed using the GraphPad Prism 5® software.

## Results

The mean values for BW, TC, and echocardiographic variables of the dogs, separated by breed, are shown in Table 1.

**Table 1:** Values (mean ± SD) for the dogs' body weight, thoracic circumference, and echocardiographic variables according to breed.

Variables				Breeds		
		DO (n=8)	LR (n=13)	GS (n=10)	MBS (n=12)	DS (n=5)
BW	(Kg)	33.25±5.14	26.31±4.52	29.25±1.90	29.25±1.90	32.20±12.97
TC	(cm)	82.38±4.71	67.00±3.18	76.10±1.59	72.83±6.02	73.00±8.63
Ao	(cm)	1.96±0.20	2.17±0.10	2.27±0.10	2.11±0.16	2.11±0.17
LA	(cm)	2.34±0.17	2.61±0.16	2.60±0.11	2.49±0.18	2.50±0.22
LA/Ao	(cm)	1.19±0.10	1.19±0.03	1.14±0.04	1.18±0.01	1.18±0.01
RVIDd	(cm)	1.72±0.14	1.73±0.45	1.83±0.15	1.64±0.31	1.64±0.31
ISTd	(cm)	0.83±0.12	0.96±0.12	1.07±0.17	1.01±0.09	1.01±0.09
LVIDd	(cm)	4.16±0.34	3.97±0.30	4.11±0.45	3.89±0.88	3.89±0.88
LVWTd	(cm)	0.77±0.12	0.85±0.11	0.93±0.10	0.98±0.13	0.98±0.13
ISTs	(cm)	1.18±0.10	1.36±0.16	1.44±0.15	1.38±0.15	1.38±0.15
LVIDs	(cm)	2.86±0.25	2.56±0.34	2.64±0.48	2.48±0.57	2.48±0.57
LVWTs	(cm)	1.22±0.10	1.31±0.13	1.27±0.15	1.30±0.17	1.30±0.17
FS	(%)	31.75±1.28	36.46±5.95	37.20±6.16	36.60±2.60	36.60±2.60
EF	(%)	72.13±2.64	71.69±3.56	72.60±4.47	70.92±6.55	68.60±1.67

MBS: Malinois Belgian Shepherd; DS: Dutch Shepherd; DO: Doberman Pinschers; GS= German Shepherd; LR= Labrador Retriever; BW= body weight; TC= thoracic circumference; Ao= aortic root dimension; LA= left atrium dimension; LA/Ao= left atrial/aortic root ratio; RVIDd= right ventricular internal dimension in diastole; ISTd= interventricular septum thickness in diastole; LVIDd= left ventricular internal dimension in diastole; LVWTd= left ventricular free wall thickness in diastole; ISTs= interventricular septum thickness in systole; LVIDs= left ventricular internal dimension in systole; LVWTs= left ventricular free wall thickness in systole; FS= fractional shortening; EF= ejection fraction.

Although Labrador Retrievers and German Shepherds presented lower BW than Dobermann Pinschers, they showed higher LA and Ao mean values. There was no significant difference in the LA/Ao values among the dog breeds. There was a positive and significant linear correlation (P = 0.0142, r = 0.6837) between BW and LA in the Malinois Belgian Shepherds.

There were no significant differences in RVIDd among the five dog breeds used in this study. Furthermore, positive correlations between BW and RVIDd were observed only in German Shepherds (P = 0.0320, r = 0.6757).

ISTd was significantly lower in Dobermann Pinschers than in Malinois Belgian Shepherds and German Shepherds; in addition, ISTS was significantly lower in Dobermann Pinschers than in any other breed. The highest mean values for both parameters were observed in German Shepherds, as detected by ANOVA and complemented by Tukey test (P < 0.05).

No significant differences were observed in the mean values of LVIDd and LVIDs among the five dog breeds. Dobermann Pinschers showed the highest mean LVID values during diastole and systole. Dutch Shepherds showed the lowest mean values in diastole, and the Malinois Belgian Shepherds showed the lowest mean values in systole. German Shepherd dogs showed a positive correlation (P = 0.0344, r = 0.6689) between BW and LVIDd. However, the same relationship was not observed for the LVIDs. In Hungarian dog breeds, BW is positively correlated with all left ventricular dimensions (Vörös et al., 2009).

The mean values of the LVWTd showed statistical differences when comparing Dobermann Pinschers with the Malinois Belgian Shepherds and Dutch Shepherds. Dobermann Pinschers had the lowest mean values, while Dutch Shepherds had the highest.

LVWTs did not differ among the breeds. However, Labrador Retrievers had the highest mean values and Dobermann Pinschers had the lowest. Furthermore, statistical differences were observed in FS between Dobermann Pinschers and Malinois Belgian Shepherds.

There were no statistical differences in LVEF among the dog breeds. However, the highest mean values were observed in German Shepherds and the lowest in Dutch Shepherds.

German Shepherds showed a positive correlation between TC and the following echocardiographic variables: RVIDd (P = 0.0307), LVIDd (P = 0.0077), and LVIDs (P = 0.0220). There was no correlation between TC and echocardiographic variables in the other evaluated dog breeds.

## Discussion

Evaluating the LA/Ao ratio through echocardiography is extremely important in detecting initial LA increases that are sometimes undetectable by radiology or electrocardiography (Boon et al., 1983; Koch et al., 1996; Lombard & Spencer, 1985).

Dogs in this study had an LA/Ao ratio slightly higher than the reference values reported by Tilley and Goodwin (2002) and corroborated by Muzzi et al. (2006) when investigating German Shepherds, and by Pellegrino et al. (2007) when studying Golden Retrievers. The rise of the LA/Ao ratio observed in this study could be a characteristic of the dog breeds studied or a cardiovascular adaptive physiological response to prolonged physical training to which those animals are subjected, as previously observed in humans (Ghorayeb et al., 2005) and horses (Bonomo et al., 2011). A higher LA/Ao ratio is considered to be accompanied by ventricular changes, such as an increase in cavity diameter or myocardial thickness. However, as diastolic function was not assessed in this study, it was not possible to determine the pressure inside the chamber. This study selected a convenient homogeneous sample of dogs for military use that were housed and trained under the same conditions. However, a larger sample of less-represented breeds would have yielded more reliable results.

According to the literature (Boon et al., 1983; Crippa et al., 1992; Gooding et al., 1986; Hanton et al., 1998; Jacobs & Mahjoob, 1988, Lombard, 1984; Sisson & Schaeffer, 1991), it is well established that RVIDd correlates directly with BW. However, only German Shepherds showed a positive linear correlation between RVIDd and BW, similar to the results reported by Morrison et al. (1992), who studied Afghan Hounds and Golden Retrievers, and by Muzzi et al. (2006), who studied German Shepherds.

In this study, the mean values for ISTd, ISTs, LVWTd, and LVWTs of the Dobermann Pinschers, Malinois Belgian Shepherds, and Dutch Shepherds were similar to those observed by Boon et al. (1983), Lombard (1984), Gooding et al. (1986), Sisson and Schaeffer (1991), and Gugjoo et al. (2014). Nevertheless, the Labrador Retrievers and German Shepherds presented slightly higher mean values than the other breeds studied, possibly because of variations in the somatotype. Furthermore, while evaluating Greyhounds, Lonsdale et al. (1998) reported an increase in ISTd, ISTs, and FS in athletic dogs compared to non-trained ones, which agrees with the results in the trained police dogs used in this study. However, it must be considered that differences in the technology or sensitivity of some echocardiography devices used in older studies can interfere with these comparisons.

The German Shepherd and Dutch Shepherd dogs assessed in this study showed a correlation between BW and LVIDd, which agrees with a previous report of the same breeds (Kayar et al., 2006). A strong correlation between BW and LVIDd has also been identified in Boxers (Cunningham et al., 2008), Border Collies (Jacobson et al., 2013), Labrador Retrievers (Gugjoo et al., 2014), and Nigerian dogs (Ajibola et al., 2017). Malinois Belgian Shepherds presented a correlation between BW and LA, and Dobermann Pinschers showed a correlation between BW and Ao. The other animals studied did not show correlations between these variables; therefore, it is possible that the differences observed could be due to somatotypes, even though there was homogeneity in the BW of dogs from the different breeds.

Muzzi et al. (2006) investigated 60 German Shepherd military dogs and reported results similar to those obtained in this study, with mean LVIDd and LVIDs of 4.17±0.50 and 3.10±0.51 cm, respectively. Jacobson et al. (2013) reported that athletic dogs, such as Border Collies, are likely to have increased LVIDs because of their minimal need for myocyte shortening for an adequate stroke volume during rest. In addition, Muzzi et al. (2006) rendered mean values for LA of 2.43 ± 0.21 cm; ISTd of 0.96 ± 0.09 cm; ISTs of 1.40 ± 0.09 cm; LVWTd of 0.88 ± 0.11 cm; and LVWTs of 1.30 ± 0.12 cm, which are similar to those observed in this study. Regardless of the dog breed, cardiac adaptations to the physical activity performed by these animals may explain the similarities found in the compared breeds.

## Conclusions

As evaluated in this study, the echocardiographic parameters of working dogs are subject to variations within the same breed and may or may not be influenced by body weight and thoracic circumference.

## References

[B001] Ajibola S., Oyewale J., Oke B., Durotoye L., Adeliyi T., Rahman S. (2017). Normal M-mode echocardiographic indices of Nigerian local dogs. *Journal of Biological Research -*. Bollettino della Società Italiana di Biologia Sperimentale.

[B002] Bonomo C. C. M., Michima L. E. S., Miyashiro P., Fernandes W. R. (2011). Quantitative echocardiography of athletic quarter horses. Ars Veterinária.

[B003] Boon J. A. (1998). Manual of veterinary echocardiography..

[B004] Boon J. A. (2011). Veterinary echocardiography..

[B005] Boon J., Wingfield W. E., Miller C. W. (1983). Echocardiographic indices in the normal dog. Veterinary Radiology.

[B006] Corda A., Pinna Parpaglia M. L., Sotgiu G., Zobba R., Gomez Ochoa P., Prieto Ramos J., French A. (2019). Use of 2-dimensional speckle-tracking echocardiography to assess left ventricular systolic function in dogs with systemic inflammatory response syndrome. Journal of Veterinary Internal Medicine.

[B007] Crippa L., Ferro E., Melloni E., Brambilla P., Cavalletti E. (1992). Echocardiographic parameters and indices in the normal Beagle dog. Laboratory Animals.

[B008] Cunningham S., Rush J., Freeman L., Brown D., Smith C. (2008). Echocardiographic ratio indices in overtly healthy boxer dogs screened for heart disease. Journal of Veterinary Internal Medicine.

[B009] Dickson D., Shave R., Rishniw M., Patteson M. (2017). Echocardiographic assessments of longitudinal left ventricular function in healthy English Springer spaniels. Journal of Veterinary Cardiology.

[B010] Ghorayeb N., Batlouni M., Pinto I. M., Dioguardi G. S. (2005). Hipertrofia ventricular esquerda do atleta. Resposta adaptativa fisiologica do coracao. *Arquivo*. Brasileiro de Cardiologia.

[B011] Gooding J. P., Robinson W. F., Mews G. C. (1986). Echocardiographic assessment of left ventricular dimensions in clinically normal English cocker spaniels. American Journal of Veterinary Research.

[B012] Gugjoo M. B., Hoque M., Saxena A. C., Shamsuz Zama M. M., Dey S. (2014). Reference values of M-mode echocardiographic parameters and indices in conscious Labrador Retriever dogs. Iranian Journal of Veterinary Research.

[B013] Hanton G., Geffray B., Lodola A. (1998). Echocardiography, a non-invasive method for the investigation of heart morphology and function in laboratory dogs: 1. Method and reference values for M-mode parameters. Laboratory Animals.

[B014] Jacobs G., Mahjoob K. (1988). Multiple regression analysis, using body size and cardiac cycle length, in predicting echocardiographic variables in dogs. American Journal of Veterinary Research.

[B015] Jacobson J. H., Boon J. A., Bright J. M. (2013). An echocardiographic study of healthy Border Collies with normal reference ranges for the breed. Journal of Veterinary Cardiology.

[B016] Kayar A., Gonul R., Or M. E., Uysal A. (2006). M-mode echocardiographic parameters and indices in the normal German Shepherd dog. Veterinary Radiology & Ultrasound.

[B017] Koch J., Pedersen H. D., Jensen A. L., Flagstad A. (1996). M‐mode echocardiographic diagnosis of dilated cardiomyopathy in giant breed dogs.

[B018] Lombard C. W. (1984). Normal values of the canine M-mode echocardiogram. American Journal of Veterinary Research.

[B019] Lombard C. W., Spencer C. P. (1985). Correlation of radiographic, echocardiographic, and electrocardiographic signs of left heart enlargement in dogs with mitral regurgitation. Veterinary Radiology.

[B020] Lonsdale R. A., Labuc R. H., Robertson I. D. (1998). Echocardiographic parameters in training compared with non‐training Greyhounds. Veterinary Radiology & Ultrasound.

[B021] Morrison S. A., Moise N., Scarlett J., Mohammed H., Yeager A. E. (1992). Effect of breed and body weight on echocardiographic values in four breeds of dogs of differing somatotype. Journal of Veterinary Internal Medicine.

[B022] Muzzi R. A. L., Muzzi L. A. L., De Araujo R. B., Cherem M. (2006). Echocardiographic indices in normal German shepherd dogs. Journal of Veterinary Science.

[B023] Pellegrino A., Petrus L. C., Pereira G. G., Soares E. C., Yamato R. J., Fernandez E. L., Larsson M. H. M. A. (2007). Padronização de parâmetros ecocardiográficos de cães da Raça Golden Retriever. Ciência Rural.

[B024] Sisson D., Schaeffer D. (1991). Changes in linear dimensions of the heart, relative to body weight, as measured by M-mode echocardiography in growing dogs. American Journal of Veterinary Research.

[B025] Snyder P. S., Sato T., Atkins C. E. (1995). A comparison of echocardiographic indices of the nonracing, healthy greyhound to reference values from other breeds. Veterinary Radiology & Ultrasound.

[B026] Tilley L. P., Goodwin J. K. (2002). Manual de cardiologia para cães e gatos.

[B027] Vörös K., Hetyey C., Reiczigel J., Czirok G. (2009). M-mode and two-dimensional echocardiographic reference values for three Hungarian dog breeds: Hungarian Vizsla, Mudi and Hungarian Greyhound. Acta Veterinaria Hungarica.

[B028] Vurucu M., Ekinci G., Gunes V. (2021). An echocardiographic study of breed-specific reference ranges in healthy French Bulldogs. Veterinary Radiology & Ultrasound.

[B029] Yamato R. J., Larsson M. H. M. A., Mirandola R. M. S., Pereira G. G., Yamaki F. L., Pinto A. C. B. C. F., Nakandakari E. C. (2006). Parâmetros ecocardiográficos em modo unidimensional de cães da raça Poodle miniatura, clinicamente sadios. Ciência Rural.

